# Navigating Stomatologic Complications Secondary to Antineoplastic Agents—A Comprehensive Review

**DOI:** 10.3390/cancers17071061

**Published:** 2025-03-21

**Authors:** Ion Alexandru Popovici, Lidia Anca Kajanto, Laura Roxana Popovici, Iolanda Georgiana Augustin, Laurentia Nicoleta Gales

**Affiliations:** 1 Department of Implant Prosthetic Therapy, Faculty of Dentistry, University of Medicine and Pharmacy “Carol Davila” Bucharest, 010221 Bucharest, Romania; ion.popovici@umfcd.ro; 2 Institute of Oncology “Alexandru Trestioreanu”, 022328 Bucharest, Romanialaurentia.gales@yahoo.com (L.N.G.); 3 Institut Gustave Roussy, 94800 Villejuif, France; 4 Oncology Department, University of Medicine and Pharmacy “Carol Davila” Bucharest, 050474 Bucharest, Romania

**Keywords:** oncologic therapy, oral toxicity, bone remodeling, mucositis, preventive dentistry, targeted agents, osteonecrosis, xerostomia, dysgeusia, interdisciplinary management

## Abstract

This review systematically examines the oral complications associated with conventional and novel anti-cancer therapies. It highlights that while molecularly targeted agents including monoclonal antibodies targeting the vascular endothelial growth factor and its receptor, the epidermal growth factor receptor, tyrosine kinase inhibitors, and immune checkpoint inhibitors tend to exhibit a lower overall toxicity profile compared to traditional cytotoxic chemotherapeutics, they are nonetheless linked to significant oral adverse events. These complications encompass inflammatory mucosal reactions known as mucositis, salivary gland dysfunction leading to a sensation of dryness in the mouth, taste alterations referred to as dysgeusia, and, critically, medication-related osteonecrosis of the jaw. In particular, bone-modifying agents such as bisphosphonates and denosumab disrupt bone remodeling and the formation of new blood vessels, thereby increasing the susceptibility to osteonecrosis of the jaw, especially following invasive dental procedures. The review delineates the multifactorial pathogenesis underlying these toxicities, which involves direct cell toxicity, impaired wound healing, and secondary infections. Furthermore, it emphasizes the importance of pre-treatment dental evaluation and preventive strategies including patient education, prophylactic dental care, and the integration of adjunctive therapies such as laser therapy and autologous platelet concentrates to mitigate these adverse effects. The analysis advocates for interdisciplinary collaboration between oncologists and dental professionals to optimize management protocols, enhance treatment adherence, and ultimately improve the quality of life for oncology patients undergoing anti-cancer therapy.

## Simple Summary

This review explains how many cancer treatments, from traditional chemotherapy to newer, targeted drugs, can cause oral adverse effects. While modern cancer therapies tend to have fewer side effects, they still often lead to inflammatory mucosal reactions, salivary gland dysfunction, taste alterations, and critically, medication-related osteonecrosis of the jaw. These complications can make eating, speaking, and keeping a good quality of life difficult for patients. The work reviews different types of cancer medications, it details how each can lead to oral issues and it also outlines strategies for preventing and managing these side effects, such as early dental checkups, proper oral hygiene, and careful timing of dental procedures before and after cancer treatment. This research is valuable because it offers clear, practical recommendations to protect the oral health of cancer patients, ultimately contributing to better treatment outcomes and quality of life.

## 1. Introduction

Conventional and novel anti-cancer agents are associated with significant toxicity. Oral complications commonly observed include mucositis, hyposalivation/xerostomia, infection, gingivitis, osteonecrosis of the jaw, discomfort, and alterations in taste perception [1,2].

As cancer treatment paradigms progress, there is increasing emphasis on developing therapies that specifically target neoplastic tissue, aiming to eradicate or control malignancies while minimizing toxic effects on non-neoplastic tissue. Molecularly targeted cancer therapies have emerged, designed to inhibit the growth and survival of cancer cells by interfering with specific molecules and pathways implicated in carcinogenesis. These therapies encompass anti-tumor monoclonal antibodies (mAbs), small molecule inhibitors, signal transduction receptor blockers, and immunotherapy. Targeted therapies are utilized in the first- and second-line treatment of various solid tumors, including malignancies of the lung, breast, kidney, colorectal region, head and neck, as well as hematologic cancers [2,3,4,5,6,7,8].

Evidence from published studies indicates that targeted cancer therapies generally result in fewer oral complications compared to conventional chemotherapy. These adverse effects are typically mild to moderate in severity. However, combining targeted therapies with conventional treatments may exacerbate the severity or duration of previously recognized toxicities. Additionally, novel adverse events not anticipated during preclinical evaluations may emerge. Oral manifestations of targeted therapies may occur independently or compound the oral complications associated with radiation and traditional chemotherapy [9].

Despite some overlap in mechanisms of action among targeted molecules, their side effect profiles can differ significantly. Some molecules affect multiple pathways, resulting in distinct signaling effects and associated toxicities. This review aims to synthesize and highlight the nature, diversity, and management of oral complications associated with anti-cancer therapies based on our experience and the available literature.

## 2. Oncology Medications with Adverse Effects on the Oral Cavity

### 2.1. Bone-Modifying Agents

#### 2.1.1. Bisphosphonates (BPs)

Bisphosphonates (BPs) are antiresorptive agents that target mainly the osteoclasts, playing a critical role in maintaining bone density and strength. These drugs are used in the prevention and treatment of several conditions, including primary and secondary osteoporosis, hypercalcemia, multiple myeloma, osteolysis due to bone metastases, and Paget’s disease [10].

BPs act on both osteoblasts and osteoclasts. In vitro studies demonstrate that BPs promote proliferation and differentiation of human osteoblast-like cells [11] while also inhibiting osteoclast function. Structurally, BPs are synthetic analogs featuring a P–C–P bond instead of the P–O–P bond found in inorganic pyrophosphates. This modification renders BPs resistant to enzymatic hydrolysis, resulting in their accumulation in the bone matrix and extremely long half-life [12]. BPs preferentially accumulate at sites of active bone resorption, facilitated by blood flow and osteoclast activity. Once incorporated into bone, BPs remain until the bone undergoes resorption, meaning bone turnover rates significantly influence BP half-life [13].

Osteonecrosis of the jaw (ONJ) is defined as a non-healing area of exposed jawbone persisting for more than eight weeks in patients receiving bisphosphonates and without a history of local radiation therapy. The disease often becomes apparent following surgical interventions, such as tooth extractions or periodontal therapy. Clinical symptoms preceding ONJ include pain, tooth mobility, mucosal swelling, erythema, and ulceration [14].

The etiology of osteonecrosis of the jaw remains uncertain. Initially termed bisphosphonate-related osteonecrosis of the jaw (BRONJ), the condition was thought to resemble radiation-induced osteonecrosis, leading to the assumption that it originated from sterile necrosis of the jawbone due to impaired blood supply. This hypothesis suggested that bisphosphonates could cause osteonecrosis by inhibiting vascular endothelial growth [15].

Later evidence proposed that ONJ does not begin as classical osteonecrosis but rather as osteomyelitis [16]. Bacterial contamination, particularly by Actinomyces and Staphylococcus, may contribute to maintaining osteomyelitic lesions. Since BP-containing bone resorbs slowly, contaminated bone may not be removed quickly enough, allowing chronic osteomyelitis to develop [17]. This hypothesis aligns with findings of similar lesions following treatment with anti-RANKL antibodies, which reduce osteoclast recruitment. Therefore, impaired bone resorption appears to be a key factor in the reduced healing capacity of these lesions [2].

The antiangiogenic role of BPs remains unclear. Possible explanations include bacterial contamination sustaining chronic osteomyelitis or reduced gingival microcirculation impairing soft tissue healing. Other contributing factors to ONJ include corticosteroid and chemotherapy use, as well as prolonged BP therapy. Longer treatment regimens are associated with a higher risk of necrosis [18].

#### 2.1.2. Denosumab

Denosumab has emerged as a groundbreaking treatment for osteoporosis, multiple myeloma (MM), and bone metastases. It has the potential to replace bisphosphonates in preventing skeletal-related events (SREs) in patients with malignant diseases [19].

Denosumab is a human monoclonal IgG2 antibody that functions as a competitive antagonist to receptor activator of nuclear factor kappa-B ligand (RANKL). It prevents RANKL from binding to its receptor, RANK, which normally promotes the proliferation and activity of osteoclasts [20]. RANK is a transmembrane receptor belonging to the tumor necrosis factor (TNF) superfamily, expressed on the surface of osteoclasts and their precursors, while RANKL is a membrane-bound protein expressed by bone marrow stromal cells (BMSCs), osteoblasts, and T-lymphocytes [21]. RANK activation is essential for osteoclast function.

Denosumab offers a more favorable adverse event profile compared to BPs, including a reduced risk of renal toxicity. Unlike bisphosphonates, RANKL inhibitors do not recycle within the body, allowing adverse effects to potentially reverse upon discontinuation. While denosumab may be a preferable option for certain cancer patients, significant adverse effects have been reported. Xgeva, a formulation of denosumab, is approved for use as a subcutaneous injection at a dose of 120 mg every four weeks to manage bone metastases caused by solid tumors. The manufacturer advises that patients take supplemental calcium (1 g daily) and vitamin D (4000 IU daily) to counteract the potential for hypocalcemia caused by denosumab [22].

Current evidence supports the growing use of RANKL inhibitors like denosumab, with a low but clinically significant risk of denosumab-related osteonecrosis of the jaw (DONJ), particularly in oncology settings. This risk may be dose-dependent, with a higher likelihood of DONJ in oncology patients compared to those treated for osteoporosis [20]. Unlike bisphosphonates (BPs), denosumab is completely eliminated from the body relatively quickly, making the necrosis risk potentially more reversible and facilitating the healing of DONJ lesions [23]. This characteristic gives denosumab a significant advantage over BPs in managing osteonecrosis and may lead to more predictable treatment outcomes for DONJ.

Denosumab’s effects on oral and maxillofacial surgery and dentistry are similar to those of BPs. Both health care providers and patients must be aware of the risk of DONJ before undergoing surgical treatment [24].

### 2.2. Chemotherapy

Chemotherapy is associated with a wide range of oral and systemic complications, many of which significantly impact patients’ quality of life. Oral mucositis is among the most common side effects, characterized by painful ulcerations and inflammation, often induced by agents such as doxorubicin, bleomycin, fluorouracil, and methotrexate [25]. Table 1 details all the oral complications due to chemotherapy.

Infectious complications frequently arise due to chemotherapy-induced neutropenia, predisposing patients to bacterial, fungal, and viral infections. Streptococcus viridans, Streptococcus mitis, and Staphylococcus aureus are commonly involved in bacterial infections, while Candida albicans, Aspergillus species, and Fusarium species contribute to fungal complications. Viral reactivations, particularly those caused by herpes simplex virus (HSV-1) and varicella-zoster virus (VZV), are also prevalent [26].

**Table 1:** Common chemotherapy agents to have adverse effects on oral cavity [27].

Oral Adverse Effect	Associated Drugs	Typical Clinical Uses
Oral mucositis/ulcerations	- Methotrexate - 5-Fluorouracil/Capecitabine - Doxorubicin/Epirubicin - Vinblastine - Cyclophosphamide - Docetaxel/Paclitaxel	- Methotrexate: Leukemias, lymphomas - 5-Fluorouracil/Capecitabine: breast, GI cancers - Doxorubicin/Epirubicin: Breast, ovarian, bladder, lung cancers - Vinblastine: Hodgkin’s lymphoma, testicular cancer - Cyclophosphamide: Breast cancer, lymphomas, leukemia - Docetaxel/Paclitaxel: Breast, prostate, lung, ovarian cancers
Xerostomia	- Methotrexate - Platinum salts - Cyclophosphamide - Docetaxel/Paclitaxel	- Platinum salts: Testicular, ovarian, bladder, lung cancers
Taste alterations (dysgeusia)	- Methotrexate - 5-Fluorouracil/Capecitabine - Platinum salts - Doxorubicin/Epirubicin - Docetaxel/Paclitaxel	
Neurotoxicity/mandibular pain	- Platinum salts - Vincristine - Vinblastine	- Vincristine: Leukemias, lymphomas, neuroblastomas
Pigmentations	- Doxorubicin/Epirubicin	
Enamel or dental defects	- Vinblastine - Cyclophosphamide	

### 2.3. Modern Molecules: Unraveling Their Impact on the Oral Cavity

A review of the literature reveals that, in recent years, an increasing number of medications have been linked to medication-related osteonecrosis of the jaw (MRONJ), although the strength of the evidence varies. It is now evident that not only bisphosphonates and denosumab can induce osteonecrosis of the jaw, but a growing list of drugs may also pose a similar risk—albeit to differing degrees [28]. Moreover, these newer agents can lead to a variety of other oral side effects. Given these concerns, it is crucial to implement preventive clinical protocols similar to those used for bisphosphonate or denosumab patients and to foster close collaboration with dental professionals to ensure early detection and effective management of any oral complications. Table 2 provides a comprehensive summary of these molecules, outlining their mechanisms of action and the associated oral side effects, as well as examples from the literature, listed in the source column.

#### 2.3.1. Monoclonal Antibodies—Anti-VEGF/VEGFR

Despite its therapeutic benefits, bevacizumab is associated with several adverse effects, including hypertension, epistaxis, gastrointestinal hemorrhage and perforation, thromboembolic events, proteinuria, impaired wound healing, and nasal septum perforation. Increasing evidence also suggests a link between bevacizumab and osteonecrosis of the jaw. ONJ following intravenous bevacizumab administration for cancer treatment was first reported by Estilo et al. in 2008 [48,49].

Delayed healing of extraction sockets, osteosclerosis, and exposed bone in the mandible accompanied by mucosal swelling were observed in patients who undergo dental procedures while they follow a chronic treatment with bevacizumab. In a comprehensive analysis by Guarneri et al. (2010), the incidence of osteonecrosis of the jaw was evaluated in patients with locally recurrent or metastatic breast cancer undergoing bevacizumab-containing therapy. The study encompassed data from three large prospective trials—AVADO, RIBBON-1, and ATHENA—involving a total of 3560 patients. The overall incidence of ONJ among patients receiving bevacizumab was found to be low, at 0.3% in the blinded phases of the two randomized trials and 0.4% in the single-arm study. However, there was a notable trend toward an increased incidence of ONJ in patients who also received bisphosphonate therapy. Specifically, in the pooled analysis of the randomized trials, the incidence of ONJ was 0.9% in patients with bisphosphonate exposure compared to 0.2% in those without. In the ATHENA study, the incidence was 2.4% in bisphosphonate-exposed patients, while no cases were reported in patients not receiving bisphosphonates [50].

In a retrospective study by Christodoulou et al. (2009), the association between the concurrent administration of bisphosphonates (BPs) and antiangiogenic agents and the risk of developing osteonecrosis of the jaw was investigated. The study reviewed data from 116 patients receiving BPs—78 with zoledronic acid and 38 with ibandronic acid—for osseous metastases from various tumors. Among these patients, 25 were also receiving antiangiogenic agents, including bevacizumab and sunitinib. ONJ developed in four patients receiving both BPs and antiangiogenic agents and in one patient receiving BPs alone. The incidence of ONJ was 16% among patients receiving both drug classes, compared to 1.1% in those treated with BPs alone, a difference that was statistically significant (*p* = 0.008). The study demonstrated a synergistic effect between BPs and angiogenesis inhibitors, such as bevacizumab, in compromising bone vascularization and wound healing. While BPs primarily inhibited osteoclast activity, reducing bone turnover and remodeling, antiangiogenic agents suppressed microvascularization, impairing the ability of bone tissues to repair and regenerate. The combination of these mechanisms likely contributed to avascular osteonecrosis, particularly in the jaw, where high bone turnover and exposure to mechanical stress further increased susceptibility [51].

Abdel-Rahman and ElHalawani investigated the association between ramucirumab-based treatments and the risk of mucosal injuries in patients with solid tumors. The study analyzed data from 11 randomized Phase II and III clinical trials. The findings indicate that patients receiving ramucirumab in combination with other treatments (mostly chemotherapy) have an increased risk of high-grade stomatitis (inflammation of the mouth) compared to those receiving control treatments. Specifically, the relative risk (RR) for all-grade stomatitis was 1.62, and for high-grade stomatitis, it was 2.72 [52].

#### 2.3.2. Monoclonal Antibodies—Anti-EGFR

Cetuximab and panitumumab are monoclonal antibodies (mAbs) targeting the epidermal growth factor receptor (EGFR), approved in multiple countries, including the U.S., Canada, and the EU, for the treatment of head and neck squamous cell carcinoma (HNSCC—cetuximab) and colorectal cancer (CRC—cetuximab and panitumumab) [53].

Oral adverse effects associated with cetuximab include mucositis, xerostomia, and bullous disorders. Cetuximab-induced mucositis typically presents as erythema with heightened sensitivity and less ulceration of the nonkeratinized mucosa compared to chemotherapy- or radiation-induced mucositis. However, in combination with cytotoxic agents, it may exacerbate ulcerative mucositis, affecting broader oral mucosal regions, including the labial mucosa [9].

The adverse effects of RT significantly impact quality of life. A meta-analysis reported increased mucositis with cetuximab plus RT compared to RT alone or RT with cytotoxic agents [54]. A study of 13 patients with HNSCC found that 31% required treatment breaks due to mucositis or skin toxicity [55].

Oral toxicities related to panitumumab are generally mild to moderate (grade 1–2). Stomatitis has been reported in 7–23% [37] of cases, while mucosal inflammation was observed in 6% (grade < 3) and 1% (grade ≥ 3) [56].

#### 2.3.3. Monoclonal Antibodies—Other Targets

Immunotherapy-related adverse effects (irAEs) have been documented in oral tissues, predominantly presenting as lichenoid and non-lichenoid lesions, as well as salivary gland dysfunction. However, the characterization of oral irAEs and their clinical implications remains insufficiently explored. Elad et al.’s retrospective clinical review analyzed 14 patients with oral irAEs, assessing their immediate effects, treatment approaches, chronicity, and potential progression to oral cancer. The most frequently reported symptoms included pain and dry mouth, ranging from mild discomfort to severe impairment of oral intake. Immediate sequelae varied from sensitivity to specific foods to complete oral intake cessation. Management strategies primarily involved conventional palliative therapies, with or without systemic corticosteroids. In six cases, discontinuation of immunotherapy was necessary. Novel treatment approaches included photobiomodulation for oral mucosal pain relief and salivary gland intraductal irrigations to alleviate salivary hypofunction. Long-term complications included the development of proliferative leukoplakia and oral cancer [57].

In their 2015 publication, Owosho et al., reported a case of osteonecrosis of the jaw associated with ipilimumab therapy. The patient, undergoing treatment for malignant melanoma, developed ONJ following the administration of ipilimumab, a monoclonal antibody that enhances immune response by inhibiting CTLA-4. This case was among the first to suggest a potential link between immune checkpoint inhibitors and ONJ, expanding the spectrum of medications implicated in this condition beyond traditional antiresorptive and antiangiogenic agents [37].

#### 2.3.4. Fusion Proteins/Decoy Receptors

In a 2016 article, Ponzetti et al. discuss a case of jaw osteonecrosis in a 64-year-old female patient undergoing treatment with aflibercept combined with irinotecan and fluorouracil for metastatic colorectal cancer. The patient, who had untreated periodontitis and a history of episodic pyorrhea, developed osteonecrosis of the jaw [58].

Another similar case was published by Zarringhalam et al. in 2017. The authors highlight the importance of monitoring the oral health of patients receiving anti-VEGF agents, due to the potential risk of developing osteonecrosis of the jaw [59].

#### 2.3.5. Small Molecule TKIs

In their 2019 systematic review, Vallina et al., examined the association between sunitinib therapy and the development of osteonecrosis of the jaw. The authors conducted a comprehensive analysis of the existing literature to assess the incidence, clinical characteristics, and potential risk factors of ONJ in patients treated with sunitinib. The review highlighted the importance of dental monitoring and preventive care in patients receiving sunitinib, emphasizing the need for further research to better understand the mechanisms underlying this association and to develop effective management strategies [60]. Table 3 summarizes all TKIs known to have oral adverse effects, the type of toxicity and frequence.

Papadopoulou et al. presented the oral side effects observed in patients with metastatic renal cell carcinoma undergoing treatment with the antiangiogenic agent pazopanib. The authors present three cases highlighting various oral complications associated with pazopanib therapy. These cases underscore the importance of dental professionals being vigilant of such adverse effects in patients receiving antiangiogenic treatments [62].

There are two case reports documenting ONJ following osimertinib therapy. Wang et al. [63] described the case of a 69-year-old woman with NSCLC who developed ONJ after four years of osimertinib monotherapy. A digital volume tomography scan revealed sequestrum formation, and she was subsequently treated with surgical debridement of the necrotic bone combined with intravenous antibiotics. A bone biopsy with subsequent histopathological analysis confirmed the diagnosis of ONJ. One week post-surgery, the wound exhibited satisfactory healing with no signs of infection. In a separate report, Subramanian et al. [64] presented the case of a 75-year-old woman who developed dental alveolar bone necrosis shortly after commencing osimertinib therapy. In this instance, the necrotic tissue was debrided via curettage until healthy, bleeding bone was exposed, and primary closure was achieved by approximating the surrounding soft tissue. The post-debridement recovery was uneventful, with rapid and uncomplicated healing.

#### 2.3.6. mTOR Inhibitors

mTOR inhibitors target the mammalian target of rapamycin and are utilized in the treatment of renal cell carcinoma (RCC), as well as for their immunosuppressive properties to prevent graft-versus-host disease following transplantation. By suppressing angiogenesis, endothelial cell proliferation, and VEGF production, rapamycin disrupts intracellular pathways involved in growth and proliferation [9].

A large multicenter, randomized, double-blind, placebo-controlled Phase III trial evaluating everolimus in patients with metastatic RCC (mRCC) following VEGF-targeted therapy identified stomatitis, rash, fatigue or asthenia, and diarrhea as the most frequently reported adverse events. Mucosal ulceration primarily affected non-keratinized oral tissues, including the labial and buccal mucosa, ventral tongue, and floor of the mouth. The term stomatitis is preferred over mucositis to differentiate mTOR-associated mucosal ulceration from mucositis induced by radiotherapy or cytotoxic chemotherapy. Stomatitis associated with mTOR inhibitors typically manifests as discrete aphthous-like ulcerations, contrasting with the broader ulcerations seen in radiation- or chemotherapy-induced mucositis. Stomatitis was observed in 40% of patients, with 3% experiencing grade 3 reactions. Mucosal inflammation of grade 2 or lower was reported in 14% of cases, while grade 3 events occurred in 1% of patients [65].

## 3. Management of Oral Cavity Adverse Effects and Complications Secondary to Oncological Treatments

### 3.1. Pre-Treatment Dental Management

The dentist plays a pivotal role in the comprehensive care of patients undergoing chemotherapy, encompassing the pre-treatment, active treatment, and post-treatment phases. Prior to any dental intervention, the oncologist should provide the dentist with a detailed overview of the patient’s medical status, underlying pathology, and prescribed antineoplastic regimen. A thorough dental assessment is imperative, including a detailed history, radiographic evaluation (periapical, bitewing, and panoramic imaging), periodontal and endodontic examination, and an assessment of existing restorations. Identifying potentially malignant oral lesions through clinical signs and symptoms is essential for early intervention [66]. Additionally, diagnostic tools such as quantitative sialometry can be valuable in predicting, evaluating, and managing potential xerostomia and hyposalivation associated with chemotherapy [67].

Patient education is a fundamental component of pre-chemotherapy dental care. Patients should be informed about the potential oral complications associated with chemotherapy, their current dental hygiene status, and the preventive and therapeutic measures required before, during, and after treatment. Denture fitting and adjustment of traumatic prostheses should be completed to prevent mucosal injury. Patients should receive comprehensive oral hygiene instructions and caries prevention strategies [68]. Prophylactic measures such as tartar removal, fluoride application, and chlorhexidine mouth rinses are highly recommended. Chlorhexidine exhibits bactericidal properties against both Gram-positive and Gram-negative bacteria by disrupting the cell membrane and enzymatic activity. A 0.12% chlorhexidine solution has been shown to effectively reduce plaque accumulation, bleeding, and Streptococcus mutans concentrations in saliva [26]. Despite these recommendations, Cardona et al., evaluated the effect of chlorhexidine on oral mucositis in cancer patients undergoing chemoradiation. Recognizing that microbial colonization of ulcerated mucosa may lead to secondary infections, they conducted a systematic review of randomized placebo-controlled trials sourced from major databases. Among the twelve studies included—nine of which were pooled in meta-analyses—chlorhexidine did not significantly reduce the incidence or severity of mucositis compared to placebo, although a trend toward significance was observed in patients receiving chemotherapy. Reported side effects included tooth staining and altered taste. The authors concluded that, based on current evidence, chlorhexidine is not sufficiently effective in preventing or mitigating oral mucositis, and further research in chemotherapy-specific populations is needed [69].

Several critical factors must be considered when formulating an individualized dental treatment plan. Although odontogenic infections are uncommon, they can lead to bacteremia in immunocompromised patients, necessitating the elimination of all potential sources of oral inflammation. Indices such as the periodontal index (PI), gingival index (GI), and the decayed, missing, and filled teeth/surfaces (DMFT/S) score provide valuable insights into the patient’s oral hygiene status and serve as predictive markers for future complications. The treatment plan must also account for the time available before chemotherapy initiation while considering the patient’s immune status and overall health [26].

Endodontic treatment should be performed in accordance with specific guidelines. In cases of reversible pulpitis, caries control is the preferred approach. For irreversible pulpitis, initial biomechanical preparation of the root canal is recommended. However, in chronic periapical conditions, endodontic treatment should be completed at least seven days before the initiation of chemotherapy. In the presence of acute periapical infections, the decision between endodontic therapy and extraction must be based on the patient’s overall health status [26]. Teeth with poor or questionable prognoses should be extracted at least 2–3 weeks before chemotherapy initiation [70]. Tooth extractions should be followed by primary wound closure. If the platelet count falls below 40,000/mm^3^, platelet transfusion is required prior to any invasive procedure. Additionally, antibiotic prophylaxis is necessary for patients with granulocyte counts below 2000/mm^3^. Ideally, minor invasive procedures should be performed at least two weeks before chemotherapy, whereas major surgical interventions should be completed 4–6 weeks before treatment initiation [67].

### 3.2. Diagnostic and Management

Dental treatment during oncologic treatment should generally be avoided unless urgent intervention is required. Invasive or traumatic dental procedures should be strictly avoided during chemotherapy. Extractions and even minor surgical interventions should not be performed due to the heightened risk of infection and impaired wound healing. Periodontal procedures such as scaling and root planing are contraindicated, as they pose a significant risk of bacterial dissemination [27].

#### 3.2.1. Xerostomia

Hyposalivation and xerostomia are frequent in oncological patients and appear often following head and neck radiation, but are also related to the use of systemic treatments with immunotherapic agents, most chemotherapy drugs, and some TKIs. Symptom management includes palliative strategies, adjusting saliva’s texture, viscosity, and acidity [71].

Xerostomia heightens infection risk and disrupts oral functions like causing difficulties in chewing, swallowing, tasting, and speaking. Also, xerostomia can cause burning, discomfort, and tongue tip erythema [72].

Management of immunotherapy-related xerostomia, defined as the subjective sensation of oral dryness, and objective dry mouth is typically achieved with the use of oral moisturizers or sialogogue agents (e.g., pilocarpine and cevimeline). Due to the increased risk of dental complications—such as caries and recurrent candidiasis infections—regular dental examinations, frequent prophylactic cleanings, and the application of topical fluoride treatments are recommended. Recommendations include rinse with 0.2% or 0.1% chlorhexidine daily to stave off infections. Patients should steer clear of tobacco and alcohol, use mouth rinses and artificial saliva, and consider pilocarpine for better chewing and swallowing comfort. Ascorbic, malic, and citric acid-rich foods can enhance saliva flow for radiation xerostomia. However, their acidity might harm oral tissues, worsen mucositis, and aid tooth demineralization [73,74].

#### 3.2.2. Osteonecrosis of the Jaw

Medication-related osteonecrosis of the jaw (MRONJ) is a severe condition resulting from adverse reactions to medications that harm the bone tissue in the mandible and maxilla. Diagnosis of MRONJ relies on established criteria by the American Association of Oral and Maxillofacial Surgeons (AAOMS), which commonly includes the presence of exposed bone or a fistula in the maxillofacial region lasting over eight weeks (Table 4). However, in 2019, the European task force on MRONJ suggested that this eight-week threshold might not be necessary for diagnosis [75,76].

Clinical evaluation and radiographic examination are fundamental for assessing necrosis and sequestrum presence. The AAOMS has defined a four-stage MRONJ classification. The pathophysiology of MRONJ is still being studied but is believed to involve factors like disruptions in bone remodeling and angiogenesis. Medications like bone-modifying agents, but also targeted therapies or immunotherapies, inhibit crucial processes in bone turnover and vascular development, contributing to bone necrosis [77].

Furthermore, dental procedures, infections, and systemic inflammation play significant roles in triggering MRONJ. It is particularly prevalent in patients with immune dysfunctions, like those with rheumatoid arthritis or diabetes, elevated by therapies like oncologic treatments and corticosteroids. Genetic factors also influence MRONJ susceptibility [78].

**Figure 1:**
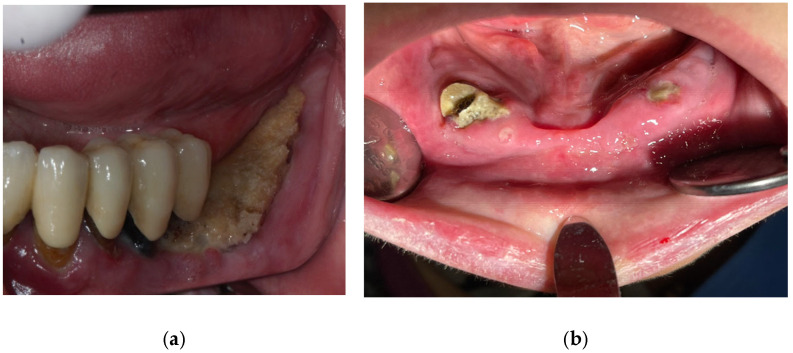
Bone sequestrum ((**a**)—left image) secondary to 15 months of Denosumab use in a 54 yo man with metastatic renal cell carcinoma treated in parallel with Nivolumab plus ipilimumab and ((**b**)—right image) secondary to 38 months of treatment with bevacizumab and 5-fluorouracil for a metastatic colon cancer in a 68 yo woman (personal archive).

**Figure 2:**
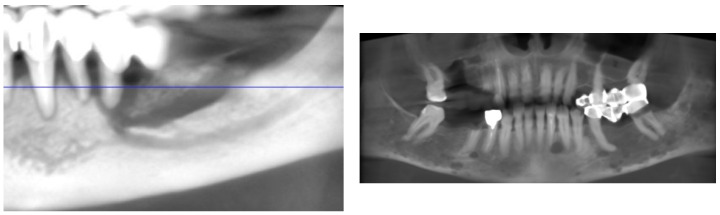
Radiological aspect of bone sequestrum (personal archive).

**Figure 3:**
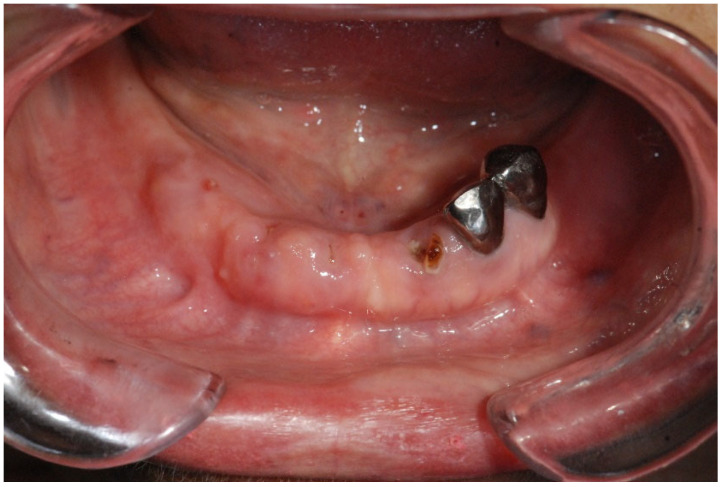
Gingival swelling (personal archive).

The risk of MRONJ increases with the dose, administration route, and duration of medications like bisphosphonates (Figure 1). Dental risk factors such as bone exostoses, periodontal disease, and tooth extractions are significant due to their influence on inflammation (Figure 2 and Figure 3) and infection, predisposing to MRONJ. Tooth extraction carries a substantial risk of MRONJ, particularly in cancer patients exposed to intravenous bone-modifying agents. Rates vary, with studies showing an incidence ranging from 0% to 6.6%, depending on the presence of metastatic bone cancer and the therapy used. An increased incidence is observed among those receiving high-dose bisphosphonates, while lower doses show much lower risks [75].

Prevention of MRONJ in this group requires a multidisciplinary approach with active patient involvement. Health care professionals should educate patients receiving denosumab (DNB) or bisphosphonates (BP) about MRONJ risks. Key preventive measures include maintaining excellent oral hygiene using fluoride products, avoiding smoking and alcohol, and scheduling regular dental checkups. Patients should also be informed about early MRONJ symptoms—such as exposed bone, jaw pain, loose teeth, pus, and non-healing sores—to enable prompt detection and management [79].

Patients scheduled to start ARDs, patients with non-restorable teeth, or those with a poor prognosis should be extracted, and any necessary elective dentoalveolar surgery should be performed. Pre- and postoperative antibiotics and antimicrobial rinses are recommended. In cases of dental infection, anti-resorptive therapy should be delayed for 45–60 days after surgery to allow proper soft tissue healing [79]. Studies indicate that following a traumatic extraction with complete osseous healing—typically requiring 4–6 weeks—the initiation of anti-resorptive therapy is safer, particularly in patients undergoing high-dose cancer therapy. While invasive procedures like implants are contraindicated because of the MRONJ risk, non-invasive procedures (e.g., restorative dentistry and endodontics) may proceed without delay [75]. Medical oncologists are encouraged to educate patients on the importance of dental health and to check for exposed bone during chemotherapy, while dentists must perform thorough oral examinations, radiographic assessments, and manage local infections prior to initiating a therapy that elevates MRONJ risk [71].

**Table 4:** Signs and symptoms of osteonecrosis of the jaw [76,78].

Sign/Symptom	Description	Stage
Gingival Swelling	Inflammation and swelling of the gums, which may occur in response to adjacent necrotic bone or local infection.	Early stages (Stage 0, progressing to Stage 2)
Dull Mandibular Pain	Persistent, non-specific pain in the mandible that may indicate early changes in bone integrity.	Stage 0
Sinus Pain	Unexplained discomfort or pain in the sinus regions, suggesting possible maxillary bone involvement.	Stage 0
Unexplained Tooth Mobility	Loosening of teeth without evident periodontal disease, possibly due to underlying bone necrosis.	Stage 0
Odontalgia	Tooth pain occurring without an obvious dental cause, potentially reflecting underlying bone pathology.	Stage 0
Exposed Bone	Visible necrotic bone in the mandible or maxilla, often detectable through an intraoral or extraoral fistula; persists for over 8 weeks.	Stage 1 and beyond
Fistula (Intraoral/Extraoral)	An abnormal tract that connects the oral cavity (or skin) to necrotic bone, allowing probing of the underlying bone tissue (Figure 4).	Stage 1+
Signs of Infection	Presence of erythema, pus discharge, and purulent drainage around exposed bone areas, indicating an active infection.	Typically Stage 2
Pathologic Fractures	Fractures of the jawbone that occur with minimal or no trauma due to significant bone compromise.	Stage 3
Extraoral Fistula	Fistula formation visible on the facial skin, reflecting advanced necrotic changes in the bone.	Stage 3
Osteolysis Beyond Alveolar Bone	Bone resorption that extends beyond the alveolar process (e.g., affecting the inferior border, ramus, sinus floor, or zygoma), indicating severe bone involvement.	Stage 3
Oroantral/Oronasal Communication	Abnormal openings between the oral cavity and the sinus or nasal areas, which may lead to further complications.	Stage 3

The primary goals of on-set MRONJ treatment are to prevent further necrosis, control infection and pain, and preserve quality of life—all while supporting ongoing oncologic treatment to manage bone pain and reduce skeletal-related events (SREs) [75]. Treatment strategies are tailored to the disease stage and include conservative (non-operative) and surgical options [80]. Conservative treatment typically involves systemic antibiotics, antimicrobial therapy, and strict oral hygiene measures, sometimes coupled with the removal of movable bony sequestra or symptomatic teeth to stabilize lesions. Although early guidelines recommended avoiding surgical interventions due to inconsistent outcomes, current evidence supports surgical treatment for patients who do not respond to conservative measures. Surgical procedures may involve debridement, sequestrectomy, or more extensive resections, with perioperative antibiotic coverage to prevent infection [75,77,78,80]. Additional treatment modalities—such as ozone therapy, laser therapy, and the use of growth factors in combination with antibiotics, as well as vitamin D supplementation for deficient patients, are possible.

Recent advancements include the use of autologous platelet concentrates (APCs) such as Platelet-Rich Plasma (PRP) and Platelet-Rich Fibrin (PRF). These APCs have been shown to reduce the onset of MRONJ and accelerate epithelization, particularly in patients on BP therapy, by modulating inflammation and enhancing immune responses. In addition, laser therapy—alone or in combination with L-PRF—has demonstrated promising results in achieving physiological wound healing and reducing MRONJ incidence [81].

Overall, the guidelines emphasize a patient-centered approach, combining meticulous preventive measures and tailored treatment strategies to reduce MRONJ risk and manage its complications effectively.

#### 3.2.3. Lichenoid Reactions/Lichen Planus

A lichenoid reaction (LR) is a pathological entity that may involve the skin, the mucosal surfaces, or both concurrently. The presentation of a lichen planus (LP) is characterized by whitish reticular papules and erythematous erosions, and, in some cases, by plaques displaying a reticular pattern with radiating striae [82]. Although the clinical appearance of LRs and LPs closely resembles that of LP, LRs are distinctly linked to a specific causative agent. Notably, an LR can resolve immediately after the agent’s effect ceases or may persist over time [83]. Furthermore, histologic evaluation typically demonstrates features such as eosinophilic infiltration, marked parakeratosis with acanthosis, and vascular inflammation surrounding the deep plexuses—findings that are not observed in LP [84].

Reaching consensus on diagnostic criteria is often challenging, in part because established LRs may persist after cessation of the offending drug unless they are rigorously managed. Nevertheless, McCartan et al., have suggested that a history of the current use of an LR-inducing medication combined with consistent histopathologic findings is likely sufficient for diagnosis, with the additional consideration that testing for circulating basal cell cytoplasmic autoantibodies may be beneficial [85].

The increasing use of biologic agents in the management of rheumatoid arthritis, ankylosing spondylitis, psoriatic arthritis, and various oncologic conditions has been accompanied by emerging reports of LRs. LRs are commonly induced by chemotherapeutic agents, targeted therapy, and even immunotherapy agents. Management of LRs primarily involves discontinuation of the offending agent. When it is not feasible to substitute or stop the causative agent, corticosteroid therapy may be employed, with oral prednisolone followed by topical corticosteroids having demonstrated satisfactory results [86].

#### 3.2.4. Pigmentations

Pigmentary alterations represent a well-documented adverse effect of imatinib mesylate, with hypopigmentation being reported far more frequently than hyperpigmentation [87]. Within the oral cavity, imatinib administration is associated with a distinct blue-gray, asymptomatic hyperpigmentation of the hard palate, which histologically corresponds to melanin deposition in the lamina propria [88]. Additionally, isolated case reports have described intraoral hyperpigmentation involving other sites, such as the gingivae and teeth. A recent multivariate analysis in a cross-sectional study of 74 participants demonstrated that the duration of imatinib therapy is directly proportional to the intensity and extent of hyperpigmentation observed, particularly in cases where hydroxyurea treatment preceded imatinib administration [89,90].

The underlying pathophysiology of these pigmentary changes, and specifically the intraoral hyperpigmentation, remains unclear. Proposed mechanisms include the direct inhibition of C-kit—a receptor normally expressed in the oral mucosa—and the deposition of drug metabolite complexes. Alternatively, hyperpigmentation may arise from the chelation of iron and melanin by a metabolite of the drug, a mechanism analogous to that observed with agents such as minocycline and chloroquine [91]. Furthermore, imatinib can lead to an overstimulation of melanin synthesis in certain cutaneous and mucosal regions. It appears that the drug interacts with multiple receptors in the skin, resulting in either the activation or inhibition of melanin production [92].

Additional medications that have been implicated in causing oral mucosal pigmentation include chemotherapy agents such as doxorubicin, docetaxel, and cyclophosphamide (affecting the tongue dorsum, buccal mucosa, and nails). In these cases, the proposed mechanism involves the stimulation of melanocytes without metabolite deposition, and notably, such pigmentation does not occur on the palatal mucosa [93,94].

Given the benign nature of these hyperpigmented lesions, treatment is generally not required. Nonetheless, laser therapy may be considered for individuals with extensive pigmentation or significant aesthetic concerns [61].

#### 3.2.5. Aphtous Ulcers/Stomatitis

Aphthous-like oral ulcers are a frequently encountered and debilitating adverse event associated with mTOR inhibitors (mTORis). In 2010, Sonis et al., introduced the term “mTOR Inhibitor Associated Stomatitis (mIAS)” to distinguish these lesions from the oral ulcerations produced by cytotoxic chemotherapy or head and neck radiotherapy—collectively referred to as “conventional oral mucositis” [95]. mIAS affects approximately 25–55% of patients and is characterized by round ulcers on nonkeratinized mucosal surfaces, typically covered by a grayish-yellow fibrin pseudomembrane with an erythematous rim [47]. Management generally involves the use of topical steroid agents, although more severe cases may require systemic steroid therapy or discontinuation of the mTORi, as mIAS is considered a dose-limiting toxicity [96].

The rapid expansion in the use of immune checkpoint inhibitors over recent years has revolutionized the treatment landscape for several hematologic malignancies and solid tumors. However, the inhibition of PD-1, PD-L1, and CTLA-4 may also trigger autoinflammatory and autoimmune responses affecting multiple organs, including the oral cavity [97]. Oral toxicities attributable to immune checkpoint inhibitors have been documented in approximately 8% of patients and are often accompanied by other systemic immunotherapy-related adverse events (irAEs). Nevertheless, the actual prevalence of oral irAEs remains uncertain due to inconsistencies in toxicity reporting [98].

Clinically, oral mucosal lesions may mimic conditions such as oral lichen planus or mucous membrane pemphigoid/bullous pemphigoid, with some cases exhibiting extensive ulceration and lip crusting similar to presentations seen in erythema multiforme or Stevens–Johnson syndrome/toxic epidermal necrolysis [99]. Management of these oral mucosal irAEs typically involves high-potency topical steroids, although systemic steroids or steroid-sparing immunosuppressive agents may be required in certain patients. In severe cases, temporary suspension of ICI therapy may be warranted until improvement or resolution of the lesions is observed [100].

The use of TNF-α inhibitors (e.g., infliximab) and IL-6 inhibitors (e.g., tocilizumab) as steroid-sparing agents in managing several irAEs further suggests that both systemic and tissue-specific cytokine levels may contribute to their pathogenesis. Indeed, the blockade of CTLA-4 and PD-1/PD-L1 leads to an increased production of cytokines—including TNF, IFN-γ, and IL-2—which can promote further T cell proliferation and activation [101,102].

#### 3.2.6. Mucositis/Gingivitis

Oral mucositis (OM) is one of the most common adverse effects of chemotherapy, manifesting as a painful inflammatory reaction of the oral mucosa. It is characterized by an initial influx of inflammatory cells, followed by disruption of the epithelium and subsequent ulceration. The initial clinical sign is erythema, often accompanied by a burning sensation. As the condition progresses, edema and ulceration ensue, impairing vital functions such as speech, swallowing, and eating. Typically, OM develops 4–7 days after the initiation of a high-dose chemotherapeutic regimen and resolves 2–4 weeks after treatment completion. Agents frequently implicated in the onset of OM include doxorubicin, bleomycin, fluorouracil, and methotrexate [103].

Risk factors for OM are multifactorial, encompassing both therapy-related and patient-specific variables. Studies indicate that higher drug doses and increased frequency of administration correlate with an elevated risk of developing OM. Moreover, the intrinsic properties of the chemotherapeutic agent contribute significantly; for instance, agents that interfere with DNA synthesis—such as antimetabolites (methotrexate, 5-fluorouracil) and purine analogs—can elevate the incidence of OM to as high as 60%. In addition, drugs like methotrexate and etoposide, which are secreted in saliva, further predispose the oral mucosa to toxicity [104]. Patient-related risk factors include advanced age, malnutrition, pre-existing medical conditions, poor oral hygiene, local trauma, liver disease, and compromised kidney function. Periodontal disease, potentially exacerbated by pathogens such as *Porphyromonas gingivalis* as well as fungal and viral organisms, also increases the risk of ulceration and mucositis [25].

Management of OM is guided by established clinical protocols. Preventative strategies emphasize stringent oral hygiene and pre-chemotherapy dental evaluation, including the use of dental floss and oral rinses. Evidence suggests that rinsing with sterile water or physiologic saline is more effective than chlorhexidine, and povidone-iodine rinses have been shown to reduce OM severity [105]. Anti-inflammatory agents, such as benzydamine, are employed for both prevention and treatment. Additional prophylactic measures include the use of histamine gels and cytoprotective drugs like amifostine, which is thought to mitigate ROS-induced damage. Antioxidants such as vitamin E and supplements like glutamine are used in both the prevention and management of OM [104]. Topical anesthetics (e.g., xylocaine, lidocaine solutions) and analgesics (e.g., morphine) are administered for pain relief. Additionally, a mouthwash formulation—comprising diphenhydramine, viscous lidocaine, bismuth subsalicylate, and corticosteroids is useful for symptomatic management [106].

Recent research has identified several interventions with proven efficacy in preventing and treating OM. Cryotherapy, which induces local vasoconstriction and thereby reduces mucosal blood flow and drug exposure, has emerged as an effective option for patients undergoing chemotherapy; however, it is contraindicated in those receiving oxaliplatin due to the risk of neurological side effects such as mandibular stiffness [107]. Application of cryotherapy 5–30 min prior to 5-fluorouracil administration may limit the severity of OM symptoms. Intravenous palifermin has also demonstrated benefits in preventing OM, and laser therapy has yielded encouraging results in both the prevention and treatment of OM among chemotherapy patients. The MASCC/ISOO 2014 guidelines recommend laser therapy with a wavelength of approximately 650 nm, a power setting of 40 mW, and a dose of 2 J/cm^2^ per square centimeter of tissue [108].

Emerging interventions continue to expand the therapeutic options for OM prevention. Zinc, for example, appears to promote wound healing and maintain epithelial integrity by enhancing re-epithelialization while also exerting anti-inflammatory and antimicrobial effects; some reports suggest that zinc sulfate may limit OM severity [109].

Management of oral lesions induced by both EGFR inhibitors and multitargeted tyrosine kinase inhibitors follows expert recommendations from the European Society of Medical Oncology, with first-line therapy consisting of basic oral care, meticulous oral hygiene, and high-potency steroids applied topically, intralesionally, or systemically [110].

#### 3.2.7. Dysgeusia

Taste disturbances are commonly observed in patients undergoing chemotherapy. Although these alterations are not life-threatening, they can cause significant discomfort and may lead to reduced food intake, potentially delaying recovery. The pathophysiology of dysgeusia is multifactorial, involving injury to specific cranial nerves (VII, IX, and X), the oral mucosa, or the taste buds [111]. Notably, taste bud dysfunction is often linked to zinc deficiency, and dysgeusia has also been associated with vitamin D deficiency [112]. Diagnostic confirmation of taste disturbances is achieved through methods such as electrogustometry, whole-mouth gustatory testing, or magnetoencephalography [113]. In various investigations, the prevalence of chemotherapy-induced dysgeusia has been reported to be approximately 39%. One study noted that 38% of patients experiencing phantogeusia and parageusia described a salty taste, while 22% reported mixed sensations (e.g., bitter-salty or sweet-sour) [114].

Chemotherapeutic agents commonly associated with taste defects include cisplatin, doxorubicin, 5-fluorouracil, docetaxel, paclitaxel, cyclophosphamide, and carboplatin [115].

Hedgehog signaling pathway inhibitors, such as vismodegib and sonidegib, are used to treat advanced, metastatic, or unresectable basal cell carcinoma. The Hedgehog pathway is crucial for the differentiation and maintenance of lingual taste receptor cells in the papillae and taste buds, ensuring proper taste function [112]. Consequently, taste alterations are one of the most common adverse events associated with these agents, with reported incidences of 55.8% for vismodegib and 44.3% for sonidegib [116]. Dysgeusia is also frequently reported in patients receiving crizotinib—a multitargeted tyrosine kinase inhibitor used for ALK-positive non-small cell lung cancer that targets ALK, MET protein, and ROS1. Although the next-generation ALK inhibitor alectinib has been associated with dysgeusia, its incidence appears lower; in one case, grade 3 dysgeusia induced by crizotinib was successfully managed by switching to alectinib [117]. Neurological adverse events have been observed in 1–12% of patients undergoing immunotherapy, and although peripheral neuropathy is relatively rare, it can manifest in the oral cavity as dysgeusia and oral dysesthesia. A recent meta-analysis indicated that the risk of dysgeusia is lower with immune checkpoint inhibitors compared to conventional chemotherapy regimens [118]. The underlying peripheral nerve damage in these immune-related events appears to be mediated by cell-mediated mechanisms, including antibody responses against compact myelin, Schwann cells, or nodal antigens, with cross-reactivity between tumor antigens and similar epitopes on healthy cells also contributing to the neurological toxicity associated with these therapies [119].

#### 3.2.8. Bleeding

Cytotoxic chemotherapeutic agents adversely affect bone marrow function, leading to thrombocytopenia and, consequently, a predisposition to excessive bleeding. Clinically, patients may present with petechiae, hematomas, or ecchymoses, with the presence of ecchymoses often serving as an indicator of a low platelet count during chemotherapy. Specifically, a platelet count below 50,000/mm^3^ contraindicates tooth extractions or other invasive procedures due to the heightened risk of hemorrhage, and counts under 20,000/mm^3^ further elevate this risk, particularly in the context of gingival inflammation. Regions such as the soft palate, floor of the mouth, lower lip, and vestibular mucosa are especially vulnerable to bleeding [120].

To minimize hemorrhagic complications, it is critical to avoid the disruption of blood clots. Various pharmacologic agents are employed to control bleeding: vasoconstrictors (e.g., epinephrine) are preferred to reduce local blood flow, while mucoadherent protectants such as cyanoacrylate can be applied to seal bleeding sites. Additionally, agents like thrombin and hemostatic collagen are used to promote clot organization and stabilization [106].

### 3.3. Post-Treatment Special Considerations

Effective management of chemotherapy-related complications is paramount. Patients should undergo regular dental evaluations—particularly during the initial months following chemotherapy initiation. Invasive procedures, such as tooth extractions, should be deferred for at least one year after treatment; however, if postponement is not feasible, several precautions must be observed. The foremost measure is the administration of antibiotic prophylaxis, which should commence 48 h prior to the procedure and continue for 7–15 days. Additionally, the use of hyperbaric oxygen therapy both before and after extractions is recommended to enhance healing. Similarly, the use of dentures should be avoided for one year; if this is impractical, the fabrication of new dentures should be postponed for 4–6 months following chemotherapy [68].

Furthermore, in patients who have received intravenous bisphosphonates, special considerations are necessary when planning dental implants, as these individuals are at a significantly higher risk of developing bisphosphonate-related osteonecrosis of the jaw—a risk that is notably different in patients who had implants placed prior to the commencement of bisphosphonate therapy. Finally, it is recommended that patients be reexamined monthly during the first three months post-treatment, then every three months during the first year, with subsequent mandatory examinations extended to every six months for up to three years [67].

## 4. Conclusions

All oncology patients should be regularly monitored for local recurrence, metastatic lesions, and the development of secondary cancers. Since dental disease susceptibility may persist lifelong after cancer therapy, the dental team must educate patients about potential long-term adverse effects and stress the importance of good oral hygiene. An individualized prevention and monitoring program should be established, with recall intervals no less frequent than every three months, at least initially.

The risk of dental caries post-cancer therapy depends on the treatment received. Salivary hypofunction from radiotherapy and chemotherapy can lead to xerostomia and a cariogenic oral environment. Additionally, altered taste, mucositis, and difficulties in mastication may prompt patients to select cariogenic foods. Poor oral hygiene—stemming from physical limitations or psychosocial distress—further increases this risk.

Periodontal health is also compromised by cancer therapy. Patients, especially those receiving bone marrow transplants with medications like ciclosporin, may develop gingival hyperplasia and require frequent hygienist support. Radiation can impair periodontal repair, leading to ligament widening and attachment loss, thereby increasing the risk of periodontal breakdown.

When restorative procedures are necessary, using materials that release fluoride is advisable. In patients with xerostomia, conservative management of cervical caries is preferred, with crowns reserved for those with excellent oral hygiene. Dental extractions in irradiated sites or in patients treated with bisphosphonates are high risk for MRONJ and should be avoided when possible. If extractions are unavoidable, prophylactic antibiotics and gentle, minimally traumatic techniques with primary soft tissue closure are recommended.

Effective dental management is critical for oncology patients, given the long-term complications associated with cancer therapy. A tailored, multidisciplinary approach can help preserve oral function, prevent disease, and enhance quality of life.

## Figures and Tables

**Figure 4:**
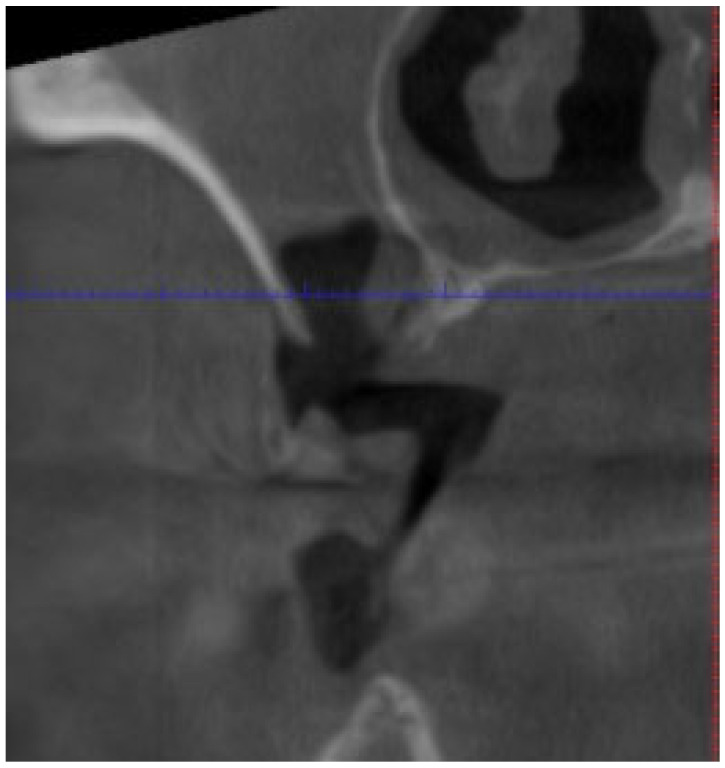
Oro-antral communication (personal archive).

**Table 2:** Comprehensive overview of molecules: mechanisms of action, clinical uses, and associated oral side effects.

Therapeutic Class	Drug	Mechanism	Common Clinical Uses	Common Oral Side Effects	Source
Monoclonal Antibodies—Anti-VEGF/VEGFR	Bevacizumab	Binds VEGF ligand to inhibit angiogenesis	Metastatic colorectal cancer, NSCLC, glioblastoma, renal cell carcinoma, cervical cancer	Stomatitis, mucositis, gingival bleeding; medication-related osteonecrosis of the jaw (MRONJ)	[29,30,31,32,33]
	Ramucirumab	Binds VEGFR2 to block VEGF signaling	Advanced gastric cancer, NSCLC, colorectal cancer	Stomatitis, mucosal irritation, bleeding, ONJ	[34]
Monoclonal Antibodies—Anti-EGFR	Cetuximab	Binds EGFR, preventing receptor activation	Metastatic colorectal cancer (KRAS wild-type), head and neck cancers	Stomatitis, mucositis, taste alterations, oral mucosal irritation	[9]
	Panitumumab	Binds EGFR, inhibiting receptor signaling	Metastatic colorectal cancer (KRAS wild-type)	Stomatitis, mucosal irritation, dryness, taste changes	[9,35]
Monoclonal Antibodies—Other Targets	Rituximab	Targets CD20 on B-cells	Non-Hodgkin’s lymphoma, CLL	Oral mucosal dryness or mild irritation; secondary infections may occur	[36]
	Ipilimumab	Inhibits CTLA-4, enhancing immune response	Metastatic melanoma (with other checkpoint inhibitors)	Stomatitis, mucosal inflammation, lichenoid reactions in the oral mucosa, MRONJ, xerostomia	[37]
Fusion Proteins/Decoy Receptors	Aflibercept	“VEGF trap” binding VEGF and placental growth factor	Metastatic colorectal cancer	Stomatitis, mucositis, MRONJ	[38]
Small Molecule TKIs	Sunitinib	Inhibits multiple kinases (VEGFR, PDGFR, c-kit, etc.)	Renal cell carcinoma, GIST, pancreatic neuroendocrine tumors	Stomatitis, mucositis, dysgeusia; MRONJ	[39]
	Lenvatinib	Multi-targeted TKI (VEGFR, FGFR, etc.)	Radioiodine-refractory thyroid cancer, renal cell carcinoma, hepatocellular carcinoma	Stomatitis, mucositis, taste alterations	[40]
	Cabozantinib	Inhibits multiple kinases (VEGFR, MET, RET, etc.)	Renal cell carcinoma, medullary thyroid cancer, hepatocellular carcinoma	Stomatitis, mucositis, dysgeusia	[41]
	Pazopanib	Inhibits VEGFR, PDGFR, c-kit	Renal cell carcinoma, soft tissue sarcomas	Stomatitis, mucositis, oral discomfort	[42]
	Axitinib	Selectively inhibits VEGFRs	Renal cell carcinoma	Stomatitis, mucosal irritation, altered taste, MRONJ	[43]
	Sorafenib	Inhibits VEGFR, PDGFR, and Raf kinases	Hepatocellular carcinoma, renal cell carcinoma, thyroid carcinoma	Stomatitis, mucositis, dry mouth and taste alterations	[9]
Small Molecule TKIs—EGFR-targeted	Erlotinib	Inhibits EGFR tyrosine kinase	NSCLC, pancreatic cancer	Stomatitis, mucositis, dry mouth, altered taste	[9]
Small Molecule TKIs—BCR-ABL	Imatinib	Inhibits BCR-ABL, c-kit, and PDGFR	Chronic myelogenous leukemia (CML), GIST, dermatofibrosarcoma protuberans	Gingival hyperplasia, mucosal pigmentation, stomatitis	[44]
Small Molecule TKIs—BTK Inhibitor	Ibrutinib	Inhibits Bruton’s tyrosine kinase (BTK)	Chronic lymphocytic leukemia (CLL), mantle cell lymphoma, Waldenström’s macroglobulinemia	Mucositis, oral bleeding, and dry mouth	[45]
mTOR Inhibitors	Everolimus	Inhibits mTOR signaling pathway	Renal cell carcinoma, HR-positive breast cancer, neuroendocrine tumors	High incidence of stomatitis and mucositis, oral ulcers, and discomfort	[46]
	Temsirolimus	Inhibits mTOR signaling pathway	Advanced renal cell carcinoma	Stomatitis, mucositis, and oral ulcerations	[47]

**Table 3:** TKIs implicated in oral toxicity [61].

TKI Name	Oral Adverse Effects	Frequency
Imatinib	Oral mucositis, gingival bleeding, taste changes	Rare
Dasatinib	Oral ulcers, stomatitis	Common
Nilotinib	Dry mouth, dysgeusia, oral pain	Uncommon
Bosutinib	Stomatitis, oral ulcers	Rare
Ponatinib	Mucositis, xerostomia, tongue pain	Rare
Erlotinib	Stomatitis, oral ulcers	Common
Gefitinib	Dry mouth, mucositis	Common
Afatinib	Stomatitis, oral ulcers	Common
Osimertinib	Oral ulcers, taste disturbances	Uncommon
Lapatinib	Stomatitis, taste changes	Uncommon
Sorafenib	Mucositis, mouth ulcers	Common
Sunitinib	Stomatitis, dysgeusia, mucositis	Common
Pazopanib	Oral ulcers, dysgeusia	Uncommon
Cabozantinib	Stomatitis, gingival bleeding	Common
Axitinib	Stomatitis, oral pain	Common
Regorafenib	Mucositis, oral pain	Common
Vandetanib	Stomatitis, dysgeusia	Uncommon
